# Supplementary material for: Comparative genomics provides new insights into the diversity, physiology, and sexuality of the only industrially exploited tremellomycete: Phaffia rhodozyma
Source: BMC Genomics. 2016 Nov 9;17:901. doi: 10.1186/s12864-016-3244-7 (PMC5103461; doi:10.1186/s12864-016-3244-7)
Supplement: Additional file 6: — List of orphan genes with links to PFAM (related to Additional file 1: Table S1). (ZIP 1428 kb) [file 12864_2016_3244_MOESM6_ESM.zip › PFAM_UNIQUE/G01670_P.html]

#                                                                            --- full sequence --- -------------- this domain -------------   hmm coord   ali coord   env coord
  
# target name        accession   tlen query name           accession   qlen   E-value  score  bias   #  of  c-Evalue  i-Evalue  score  bias  from    to  from    to  from    to  acc description of target
  
#------------------- ---------- ----- -------------------- ---------- ----- --------- ------ ----- --- --- --------- --------- ------ ----- ----- ----- ----- ----- ----- ----- ---- ---------------------
  
G01670\_P             -            944 bZIP\_2               PF07716.10    54     0.064   11.8   1.6   1   1   0.00082      0.23   10.0   1.6    24    54    43    73    40    73 0.95 - Basic region leucine zipper  
G01670\_P             -            944 DivIC                PF04977.10    80     0.024   12.8   1.9   1   1   0.00023     0.052   11.7   1.9    17    55    44    81    36    89 0.86 - Septum formation initiator  
G01670\_P             -            944 DUF4472              PF14739.1    108     0.033   13.3   1.1   1   1   9.8e-05      0.12   11.5   1.1    21    68    45    91    35   116 0.76 - Domain of unknown function (DUF4472)  
G01670\_P             -            944 DUF972               PF06156.8    107     0.014   14.4   1.9   1   1   0.00016     0.037   13.1   1.6    14    59    43    88    30   109 0.78 - Protein of unknown function (DUF972)  
G01670\_P             -            944 ERM                  PF00769.14   246         8    4.6   8.0   1   2     0.027       3.9    5.6   0.0    77   133    46    99    28   145 0.80 - Ezrin/radixin/moesin family  
G01670\_P             -            944 ERM                  PF00769.14   246         8    4.6   8.0   2   2       3.3   4.9e+02   -1.3   5.5    45   182   697   831   679   845 0.55 - Ezrin/radixin/moesin family  
G01670\_P             -            944 Myc\_N                PF01056.13   329     0.002   16.0  11.6   1   2   8.2e-06     0.002   16.0  11.6   143   246   713   821   639   848 0.75 - Myc amino-terminal region  
G01670\_P             -            944 Myc\_N                PF01056.13   329     0.002   16.0  11.6   2   2      0.51   1.2e+02    0.3   1.0   210   272   832   891   821   902 0.48 - Myc amino-terminal region  
G01670\_P             -            944 Shugoshin\_C          PF07557.6     26   2.1e-08   32.2   2.9   1   2         2     6e+03   -7.2   6.4     3    10   634   641   632   642 0.57 - Shugoshin C terminus  
G01670\_P             -            944 Shugoshin\_C          PF07557.6     26   2.1e-08   32.2   2.9   2   2   6.9e-12   2.1e-08   32.2   2.9     5    26   696   718   695   718 0.95 - Shugoshin C terminus  
G01670\_P             -            944 Sigma70\_ner          PF04546.8    211       6.5    5.0  14.0   1   2     0.042         6    5.1   0.3    21    67   576   646   566   715 0.64 - Sigma-70, non-essential region  
G01670\_P             -            944 Sigma70\_ner          PF04546.8    211       6.5    5.0  14.0   2   2      0.28        39    2.4   7.9    41    82   795   836   779   873 0.62 - Sigma-70, non-essential region  
G01670\_P             -            944 XAP5                 PF04921.9    239       2.3    6.4   5.9   1   2       3.5   3.5e+03   -4.0   0.0    13    30   620   634   611   662 0.58 - XAP5, circadian clock regulator  
G01670\_P             -            944 XAP5                 PF04921.9    239       2.3    6.4   5.9   2   2     0.002         2    6.6   4.1     8    79   792   863   787   873 0.56 - XAP5, circadian clock regulator
